# The Effects of Medium-Chain Triglyceride Oil and Butter on Lipid Profiles

**DOI:** 10.7759/cureus.62556

**Published:** 2024-06-17

**Authors:** Thanh D Hoang, Jennifer S Hatfield, Karl Nadolsky, Osei Bonsu, Priti V Nath, Francois O Tuamokumo, Mohamed K Shakir

**Affiliations:** 1 Endocrinology, Walter Reed National Military Medical Center, Bethesda, USA; 2 Endocrinology, Michigan State University College of Human Medicine, Grand Rapids, USA; 3 Research Program, Walter Reed National Military Medical Center, Bethesda, USA

**Keywords:** coffee energy drinks, clarified butter, lipids, coffee, mct oil

## Abstract

Background and objective

Butter coffee drinks, mainly a form of a saturated fat diet, are widely accepted as a "healthy energy-boosting drink", especially in the young and healthy military population. The objective of our study was to determine the effects of medium-chain triglyceride (MCT) oil and butter on lipid profile, especially apolipoprotein B (ApoB), low-density lipoprotein (LDL)-cholesterol (LDL-C), high-density lipoprotein (HDL)-cholesterol (HDL-C), and other risk factors for coronary heart disease, such as BMI, BP, fasting blood glucose, HbA1c, and high-sensitivity C-reactive protein (hs-CRP) levels in healthy adults.

Materials and methods

We conducted a prospective study of 60 subjects who were randomized to one of the two following regimens: (1) coffee or (2) coffee with butter plus MCT oil combination. The primary outcome was the effect on ApoB. Secondary outcomes were as follows: non-HDL-C, LDL-C, triglycerides, BP, waist circumference, fasting blood glucose, and HbA1c. These parameters were evaluated at the baseline and after 12 weeks. The Mann-Whitney U test was utilized for analysis of the results.

Results

While 60 subjects were recruited for the study, only 41 completed it, meeting the minimum required sample size (17 per group) necessary to achieve the desired effect size: 21 males (nine in the control group and 12 in the experimental group) and 20 females (10 in each group). Anthropometric measures were similar between the two groups at baseline, and so were age and BMI (average age: 33.00 ± 5.84 years among controls and 30.86 ± 6.14 years in the experimental group; BMI: 27.35 ± 4.63 kg/m^2^ vs. 25.74 ± 2.70 kg/m^2^). The pulse rate was 69.35 ± 10.98 in the control vs. 70.68 ± 10.32 bpm in the experimental group. The waist size was also similar in both groups. Baseline lab findings were as follows: ApoB: 89.85 ± 17.52 (control), 81.60 ± 12.84 mg/dL (experimental); hs-CRP: 0.18 ± 0.27 (control), 0.17 ± 0.27 mg/L (experimental); LDL-C 113.65 ±23.71 (control), 106.50 ± 18.99 mg/dL (experimental); HDL-C 57.35 ± 14.63 (control), 62.41 ± 16.15 mg/dL (experimental); and triglycerides: 76.00 ± 31.30 (control), 56.77 ± 14.77 mg/dL (experimental), and these values were similar. The values after 12 weeks of intervention were as follows: BMI: 27.37 ± 5.24 (control), 26.36 ± 3.55 (experimental); pulse rate: 78.88 ± 14.00 (control), 74.20 ± 11.90 bpm (experimental); ApoB 87.1 ± 17.38 (control), 85.7 ±20.59 mg/dL (experimental); hs-CRP 0.26 ± 0.22 (control), 0.15 ± 0.14 mg/L (experimental); LDL-C 111.59 ± 20.35 (control), 114.10 ± 26.99 mg/dL (experimental); HDL-C 57.71 ± 12.93 (control), 64.85 ± 13.32 mg/dL (experimental); and triglycerides: 74.71 ± 25.39 (control), 60.80 ± 15.77 mg/dL (experimental).

Conclusion

At a significance level of 5%, there was no difference between the two groups, either at the baseline or at 12 weeks of intervention. Based on our findings, adding MCT oil and butter to coffee may be safe. However, further studies with larger sample sizes and longer duration are needed to validate our findings.

## Introduction

While high saturated fat consumption has been traditionally associated with adverse cardiovascular outcomes, recent data do not suggest a clearly defined direct link between the two [1-3]. Due to its alleged health benefits, there has been renewed interest in consuming organic grass-fed cow butter and supplementation with medium-chain triglyceride (MCT) oils or coconut oils (the main source of MCTs), especially as part of butter coffee drinks. The advocates of high-fat diets in the health and wellness communities have pioneered this movement. The reported benefits of butter coffee drinks include satiety with weight loss, increased energy, improved mental function, and cardiometabolic improvements of lipoproteins, inflammation, and insulin sensitivity. This saturated fatty acid (SFA) diet contrasts with many mainstream federal health and medical dietary recommendations, including professional medical organizations and the Federal Food and Drug Administration [4,5]. However, evidence suggests that replacing long-chain SFAs and carbohydrates with sources of dietary fat such as monounsaturated fats (MUFAs), polyunsaturated fats (PUFAs), and MCT SFAs, have relatively favorable effects on lipoprotein and cardiovascular disease (CVD) outcomes [6,7].

Butter is comprised of a large proportion of long-chain SFA including 31% palmitic acid, 12% myristic acid, and 11% stearic acid. Palmitic and myristic acids have greater hypercholesterolemic effects than stearic acids [8]. In previous studies, butter consumption has been associated with an increase in serum low-density lipoprotein (LDL) concentration [9]. LDL and apolipoprotein B (ApoB)-containing lipoproteins are unequivocally the principal risk factors for the development of atherosclerotic cardiovascular disease (ASCVD) and are influenced by many other cardiometabolic factors [10]. Therefore, reducing the levels of LDL, non-high-density lipoprotein (HDL), and ApoB is recommended based on individuals' ASCVD risk [11,12]. MCTs are straight-chain SFAs that have a chain length of 6-12 carbon atoms [5]. MCT oil, produced by the fractionation of coconut oil, contains mostly caprylic and capric acids. Evidence supports the potential advantages of MCT oil over other isocaloric fatty acid sources and carbohydrates for improving appetite, thermogenesis, and weight loss [13-15]. 

‘‘Bulletproof coffee’’ is a combination of black coffee and grass-fed butter with caprylic acid (brain octane oil) and is widely promoted as a ‘‘healthy’’ energy-boosting beverage that leads to improved concentration and weight loss. The common ingredients of ‘Bulletproof coffee include two tablespoons of unsalted grass-fed butter, and one tablespoon of brain octane oil mixed with one to two cups of branded ‘‘Bulletproof Upgraded Coffee”. This has become a popular drink among US military personnel. In line with the health effects of coffee, the trending beverage "Bulletproof coffee" [16] has gained the attention of the lay press. In summary, Bulletproof coffee is generally a mixture of coffee, pasture organic butter, and coconut oil. Occasionally, MCTs (as the only fat source) are frequently added to coffee. However, its effects have not been thoroughly investigated.

In this study, we evaluated the effects of adding butter and/or MCT oil ("Bulletproof coffee") to the baseline diet of healthy adults to determine whether this trend has any adverse effects. We used MCT oil instead of brain octane oil.

## Materials and methods

MCT oil and Kerrygold® butter were obtained from Nestle Health Science (nestlenutritionstore.com) and Igourmet (opensky.com), respectively. In this study, we used MCT oil from Nestle Health Science in place of brain octane oil since we could not purchase brain octane oil from a reliable manufacturing company. The dietary composition of the control and experimental subjects was assessed by a recall diary closely monitored by a nutritionist. The diet composition - carbohydrate 50-60%, fat 25-30%, and protein 15-20% - were similar in both groups during the study period. Additionally, the study subjects were instructed to maintain a consistent dietary regimen four weeks before the initiation of the study and to continue restricted dietary regimens throughout the study.

**Figure 1:**
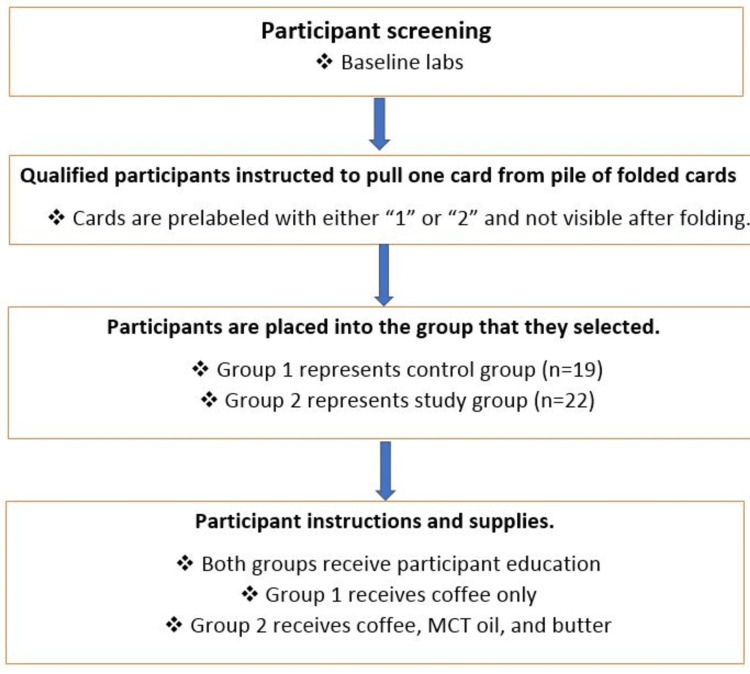
The randomization process The study subjects were randomized via a computer-generated random number of listings to receive one of two different treatment regimens. Sixty participants were recruited for the study, and 41 participants (19 in the control group and 22 in the experimental group) completed the study. Group 1 (control) was given coffee with <50 kcal of creamer and/or sweetener. Group 2 (experimental) was given coffee with Kerrygold® butter and MCT oil. The study period lasted for a total of 12 weeks MCT: medium-chain triglyceride

All laboratory tests were performed at our medical center or Lab CorpTM (Burlington, NC). This prospective randomized study included 60 participants. The participants were randomized via computer-generated random number listings to receive one of two different dietary treatment regimens (Figure 1). Group 1 was given coffee with <50 kcal of creamer and/or sweetener vs. Group 2 was given coffee with Kerrygold® butter from grass-fed cows (two tablespoons added daily, equaling 22 g fat) + MCT oil (two tablespoons added daily, equaling 28 g fat). This was added to their baseline diets via coffee as a vehicle. The doses of butter and MCT oil were gradually titrated (one tablespoon of butter + one tablespoon of MCT for five days, two tablespoons of butter + one tablespoon of MCT for five days, and finally, two tablespoons of butter + two tablespoons of MCT oil divided into three potions and added to coffee for 12 weeks. Subjects in the control group (Group 1) continued to have coffee with <50 kcal of creamer and/or sweetener for a similar period. Both control and experimental group subjects consumed plain coffee or coffee with butter + MCT oil on three occasions: 0700-0900, 1100-1300, and 1600-1700 hours daily respectively for 12 weeks.

All participants (control group and experimental group) underwent laboratory tests in the fasting state at baseline and after 12 weeks of treatment. The laboratory tests included fasting lipids with ApoB, thyroid-stimulating hormone (TSH), comprehensive metabolic panel (CMP), fasting glucose, HbA1c, and high-sensitivity C-reactive protein (hs-CRP). 

The Mann-Whitney U-test was used to compare the results at the baseline and after 12 weeks post-treatment. Participants underwent a clinical evaluation comprising a focused history of dietary intake and a review of current medications (over-the-counter medications, supplements, and vitamins), and anthropometric measurements [height (cm), body weight (kg), and waist circumference (cm)].

Inclusion criteria

Age between 18-45 years; serum levels of LDL-cholesterol (LDL-C) <160 mg/dL, non-HDL-cholesterol (HDL-C) <190 mg/dL, and ApoB <120 mg/dL; and willingness to drink three to four cups of regular caffeinated coffee daily.

Exclusion criteria

BMI over 30 or <20 kg/m^2^; waist circumference >102 (males) or 88 cm (females); triglycerides >150 mg/dL; hypertension (systolic blood pressure >150 mmHg, diastolic blood pressure >90 mmHg, or on treatment for hypertension); chronic liver disease (clinical history or a history of serum AST or ALT >3 times the upper limit of normal); renal disease (GFR <60 ml/min or history of nephritic syndrome); impaired glucose metabolism (HbA1c ≥5.7%, fasting blood glucose ≥100 mg/dL, or two-hour OGTT blood glucose ≥140-199 mg/dL); malignancy; known malabsorption disorder such as inflammatory bowel disease, celiac disease, cystic fibrosis, history of gastric bypass; hypothyroidism per lab evaluation at baseline; pregnancy; PCOS or irregular menstrual periods; on medications such as glucocorticoids, immunosuppressants (cyclosporine, sirolimus, etc.), tamoxifen, androgens, antipsychotics, hydrochlorothiazide, retinoids, beta-blockers, statins, bile-acid sequestrants, niacin, fibrates, ezetimibe, high dose fish oil (>1 g/day EPA + DHA or DHA), or any other supplements/pharmacologic agents known to alter lipoproteins (if on hormonal contraceptives, lipid panel must be stable over the past 12-24 months); Cushing’s syndrome per medical history or clinical suspicion; HIV per medical history; chronic inflammatory disorders, including but not limited to, systemic lupus erythematosus, rheumatoid arthritis, inflammatory bowel disease; history of tobacco use in the last 12 months (cigarettes, e-cigarettes, chewing tobacco, cigars, and pipes); history of marijuana use in the last 12 months; active intentional weight loss of more than 5% over the past three months.

The inclusion and exclusion criteria are presented in Table 1.

**Table 1:** Inclusion and exclusion criteria details ALT: alanine transaminase; AST: aspartate aminotransferase; BMI: body mass index; DHA: docosahexaenoic acid; EPA: eicosapentaenoic acid; GFR: glomerular filtration rate; HDL-C: high-density lipoprotein cholesterol; HIV: human immunodeficiency virus; LDL-C: low-density lipoprotein cholesterol; OGTT: oral glucose tolerance test

Exclusion criteria	Inclusion criteria
BMI over >30 kg/m^2^ or <20 kg/m^2^; waist circumference >102 cm (males) or 88 cm (females)	Age between 18-45 years
Serum triglycerides >150 mg/dL	Serum levels of LDL-cholesterol <160 mg/dL, non-HDL-cholesterol <190 mg/dL, and apo B <120 mg/dL
Hypertension (systolic blood pressure >150, diastolic blood pressure >90, or treatment for hypertension)	Willingness to drink 3-4 cups of regular caffeinated coffee daily
Chronic liver disease (clinical history or a history of serum AST or ALT >3 times the upper limit of normal)	
Renal disease (GFR <60 ml/min or a history of nephritic syndrome)	
Impaired glucose metabolism (HbA1c ≥5.7, fasting blood glucose ≥100 mg/dL, or 2-hour OGTT blood glucose ≥140-199 mg/dL)	
Malignancy	
Known malabsorption disorders including inflammatory bowel disease, celiac disease, cystic fibrosis, or a history of gastric bypass	
Hypothyroidism per lab evaluation at baseline	
Pregnancy	
Polycystic ovary syndrome or irregular menstrual periods	
On medications such as glucocorticoids, immunosuppressants (cyclosporine, sirolimus, etc.), tamoxifen, androgens, antipsychotics, hydrochlorothiazide, retinoids, beta-blockers, statins, bile-acid sequestrants, niacin, fibrates, ezetimibe, high dose fish oil (>1 g/day EPA + DHA or DHA), or any other supplements/ pharmacologic agents known to alter lipoproteins (if on hormonal contraceptives, serum lipid panel must be stable over the past 12-24 months)	
Cushing’s syndrome per medical history or clinical suspicion	
HIV per medical history	
Chronic inflammatory disorders, including but not limited to systemic lupus erythematosus, rheumatoid arthritis, inflammatory bowel disease	
History of tobacco use within the previous 12 months (cigarettes, e-cigarettes, chewing tobacco, cigars, and pipes)	
History of marijuana use within the previous 12 months	
Active intentional weight loss of more than 5% over the past 3 months	

In total, 60 participants were recruited for the study, 41 of whom completed the study. The groups included 22 participants (10 females and 12 males) in the butter and MCT oil groups, and 19 (10 females and nine males) in the plain coffee group. The study protocol was approved by our institutional review board. Informed consent was obtained from all study subjects.

## Results

A total of 60 participants were recruited (Figure 1) for the study; 41 participants (19 in the control group and 22 in the experimental group) completed the study. This study met the minimum sample size (17 participants per group) necessary to detect the desired effect. The control group consisted of nine males and 10 females. In this group, the mean age was 33.00 ± 5.84 years and the baseline BMI was 27.35 ± 4.63 kg/m^2^. The experimental group included 12 males and 10 females.

Anthropometric measures were similar between the two groups at baseline. Age and BMI were similar between the control and experimental groups (average age: 33.00 ± 5.84 years in the control and 30.86 ± 6.14 years in the experimental group; BMI: 27.35 ± 4.63 kg/m^2^ in the control group vs. 25.74 ± 2.70 kg/m2 in the experimental group). The pulse rate was 69.35 ± 10.98 beats per minute in the control vs. 70.68 ± 10.32 bpm in the experimental group. Baseline lab findings were as follows: ApoB: 89.85 ± 17.52 mg/dL (control), 81.60 ± 12.84 (experimental); hs-CRP: 0.18 ± 0.27 mg/L (control), 0.17 ± 0.27 mg/L (experimental); LDL-cholesterol: 113.65 ± 23.71 mg/dL (control), 106.50 ± 18.99 mg/dL (experimental); HDL-cholesterol: 57.35 ± 14.63 mg/dL (control), 62.41 ± 16.15 mg/dL (experimental); and triglycerides: 76.00 ± 31.30 mg/dL (control), 56.77 ± 14.77 mg/dL (experimental).

The findings at 12 weeks after intervention were as follows: BMI: 27.37 ± 5.24 kg/m2 (control), 26.36 ± 3.55 kg/m2 (experimental); pulse rate: 78.88 ± 14.00 bpm (control), 74.20 ± 11.90 bpm (experimental); ApoB: 87.1 ± 17.38 mg/dL (control), 85.7 ± 20.59 mg/dL (experimental); hs-CRP: 0.26 ± 0.22 mg/L (control), 0.15 ± 0.14 mg/L (experimental); LDL-cholesterol: 111.59 ± 20.35 mg/dL (control), 114.10 ± 26.99 mg/dL (experimental); HDL-cholesterol: 57.71 ± 12.93 mg/dL (control), 64.85 ± 13.32 mg/dL (experimental); and triglycerides: 74.71 ± 25.39 mg/dL (control), 60.80 ± 15.77 mg/dL (experimental) (Table 2).

**Table 2:** Anthropometric values and vital signs in controls (n=19) and experimental subjects (n=22) at baseline and after 12 weeks of intervention There were no differences in age, BMI, pulse rate, and blood pressure between the two groups both under basal conditions and after 12 weeks of intervention (>0.05). The Mann-Whitney U test was used to compare the results BMI: body mass index; SD: standard deviation

Variable	Baseline (control), mean ± SD	Baseline (experimental), mean ± SD	12-week (control), mean ± SD	12-week (experimental), mean ± SD
Age (years)	33.00 ± 5.84	30.86 ± 6.14	N/A	N/A
Height (cm)	174.42 ± 7.83	170.83 ± 10.95	174.42 ± 7.83	170.83 ± 10.95
Weight (kg)	82.91 ± 17.68	74.21 ± 12.47	82.18 ± 18.92	76.10 ± 13.99
Waist (cm)	87.99 ± 13.93	81.01 ± 7.22	86.69 ± 9.36	83.23 ± 8.97
BMI (kg/m^2^)	27.35 ± 4.63	25.74 ± 2.70	27.37 ± 5.24	26.36 ± 3.55
Pulse (bpm)	69.35 ± 10.98	70.68 ± 10.32	78.88 ± 14.00	74.20 ± 11.90
Systolic blood pressure (mmHg)	121.65 ± 12.29	113.77 ± 12.04	115.00 ± 14.44	113.25 ± 10.70
Diastolic blood pressure (mmHg)	74.85 ± 6.97	70.86 ± 11.78	72.76 ± 9.13	68.25 ± 9.70

**Figure 2:**
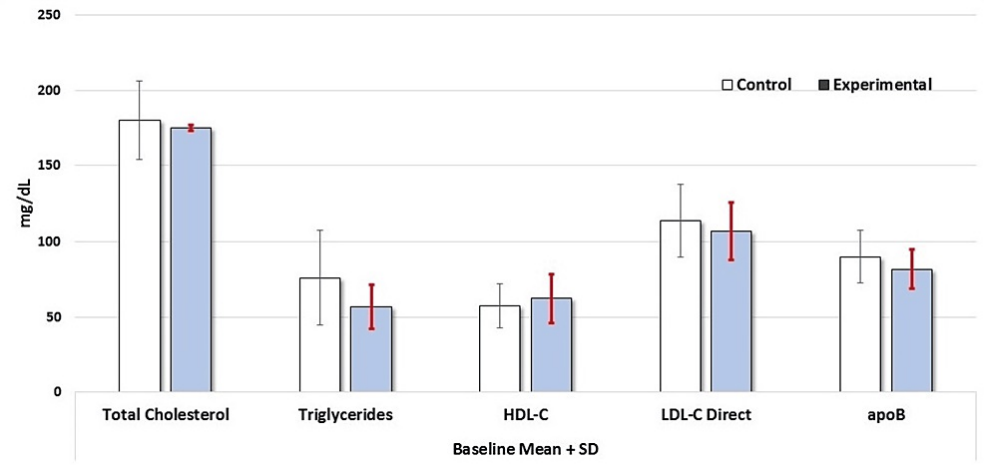
Serum levels of total cholesterol, triglycerides, HDL-C, LDL-C, and ApoB levels in control and experimental groups at baseline There were no differences in any of the laboratory values (p>0.05). The Mann-Whitney U test was used to compare the results ApoB: apolipoprotein B; HDL-C: high-density lipoprotein cholesterol; LDL-C: low-density lipoprotein cholesterol

**Figure 3:**
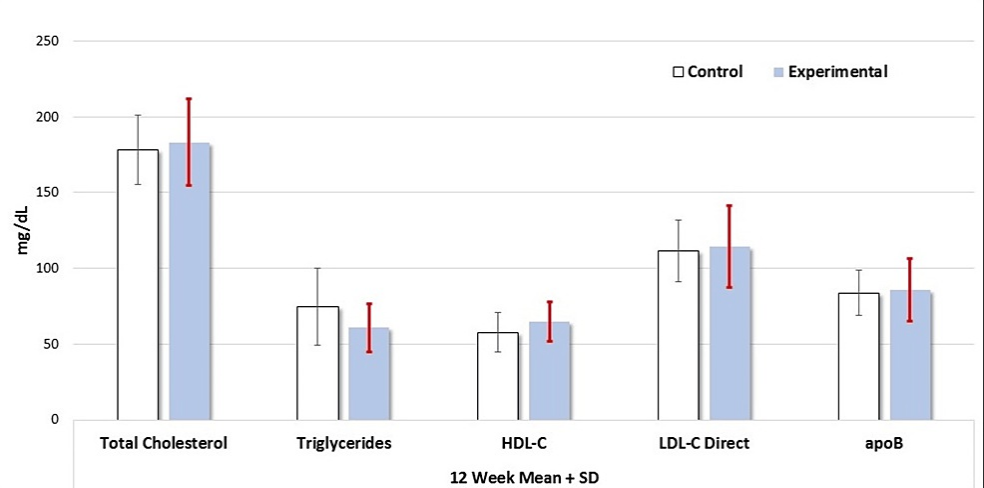
Serum levels of total cholesterol, triglycerides, HDL-C, LDL-C, and ApoB levels in control and experimental groups after 12 weeks of intervention There were no differences in any of the laboratory values (p>0.05). The Mann-Whitney U test was used to compare the results ApoB: apolipoprotein B; HDL-C: high-density lipoprotein cholesterol; LDL-C: low-density lipoprotein cholesterol

Table 3 shows a comparative analysis of serum levels of glucose, HbA1c, TSH, lipid hs-CRP, and ApoB between the control and experimental groups at baseline after 12 weeks. The serum glucose values were similar at baseline (89.45 ± 7.51 mg/dL) and at the end of 12 weeks (91.19 ± 9.82 mg/dL) in the control group. There was also no difference in the mean glucose values between baseline and at 12 weeks (88.55 ± 7.42 vs. 87.05 ± 6.13 mg/dL) in the experimental group. The serum TSH levels remained unchanged in both groups at baseline and the end of the 12 weeks. The serum cholesterol and triglyceride levels were also similar in both groups at baseline and at 12 weeks (Figures 2-3). The baseline serum HDL level in the control group was 57.35 ± 14.63 mg/dL at baseline and there was no significant change in HDL levels in this group at the end of 12 weeks (62.41 ± 16.15 mg/dL). The baseline HDL level in the experimental group was 57.71 ± 12.93 and the value was 64.85 ± 13.52 at the end of 12 weeks (not statistically significant). There were no changes in serum LDL, non-HDL, total cholesterol/HDL ratio, or ApoB levels between basal state and post-12 weeks in both control and experimental subjects (Figures 2-3). As for the serum hs-CRP level, it was 0.18 ± 0.27 mg/L in the control group at baseline and there was no change in these values at the end of 12 weeks (0.17 ± 0.27 mg/dL). In the experimental group, serum hs-CRP level also remained unchanged after 12 weeks of butter coffee therapy (Table 3).

**Table 3:** Serum levels of glucose, HbA1c, TSH, lipid, hs-CRP, and ApoB in the control (n=19) and experimental subjects (n=22) at baseline and at the end of 12 weeks There were no differences in any of these laboratory values (>0.05). The Mann-Whitney U test was used to compare the results ApoB: apolipoprotein B; BMI: body mass index; hs-CRP: high-sensitivity C-reactive protein; HbA1c: hemoglobin A1c; HDL-C: high-density lipoprotein cholesterol; LDL-C: low-density lipoprotein cholesterol

Variable	Baseline (control), mean ± SD	Baseline (experimental), mean ± SD	12-week (control), mean ± SD	12-week (experimental), mean ± SD
Glucose (mg/dL)	89.45 ± 7.51	88.55 ± 7.42	91.19 ± 9.82	87.05 ± 6.13
HbA1c (%)	5.20 ± 0.27	5.03 ± 0.27	5.25 ± 0.30	5.18 ± .27
TSH (mIU/L)	2.18 ± 0.84	2.01 ± 0.85	1.89 ± 0.69	2.10 ± 1.08
Total cholesterol (mg/dL)	180.12 ± 26.29	174.82 ± 1.97	178.24 ± 22.97	183.20 ± 28.73
Triglyceride (mg/dL)	76.00 ± 31.30	56.77 ± 14.77	74.71 ± 25.39	60.80 ± 15.77
HDL-C (mg/dL)	57.35 ± 14.63	62.41 ± 16.15	57.71 ± 12.93	64.85 ± 13.32
LDL-C direct (mg/dL)	113.65 ± 23.71	106.50 ± 18.99	111.59 ± 20.35	114.10 ± 26.99
non-HDL-C (mg/dL)	120.20 ± 30.67	112.50 ± 25.51	120.53 ± 23.62	118.30 ± 29.54
Total cholesterol/HDL ratio	3.31 ± 0.82	3.25 ± 2.36	3.22 ± 0.77	2.93 ± 0.76
hs-CRP (mg/L)	0.18 ± 0.27	0.17 ± 0.27	0.26 ± 0.22	0.15 ± 0.14
ApoB (mg/dL)	89.85 ± 17.52	81.60 ± 12.84	87.1 ± 17.38	85.7 ± 20.59

## Discussion

This study was conducted to evaluate the effects of adding MCT oil and butter to the diet among healthy subjects to determine whether there were any adverse changes in lipoproteins since butter coffee drinks ("Bulletproof coffee") are widely used, especially in the military population. No statistically significant differences were found between the two groups at baseline or during the interval from baseline until 12 weeks.

The dairy industry has provided evidence to support the beneficial role of dietary products in health including a reduced risk of heart disease and diabetes mellitus [17-19]; however, those studies have not focused on concentrated high-fat dietary forms such as butter and cream [20]. Toklu et al. [21] studied the effects of Bulletproof coffee in a patient with dyslipidemia and noted increased serum LDL-c levels; they concluded that the increased LDL-c levels were due to the diet enriched in saturated fat, mainly resulting from the incorporation of “Bulletproof coffee” into the diet. However, a recent study compared the butter from grass-fed cows to conventional Danish butter [22] and showed that a eucaloric replacement of fat type from the diet for three months had no significant change in serum lipoprotein levels between the two groups. Including butter obtained from grass-fed cows in the diet did not significantly affect the lipoprotein profiles in our study, despite the butter being co-administered with MCT oil. Another recent trial compared butter to coconut oil and olive oil and showed that using butter increased non-HDL levels compared to coconut oil, whereas coconut oil did not significantly differ from olive oil [23].

However, the effects and potential benefits of MCT oil on lipoproteins are unclear. A review of studies on MCT oil in 2002 concluded that there may be mildly improved energy expenditure, and a potential calorie deficit from improved satiety when MCT oil is used to iso-calorically replace full-form long-chain triglycerides (LCT) [15]. One study among patients with hypertriglyceridemia and a BMI of 24-28 kg/m^2^ showed that consumption of MCT oil reduced waist circumference and serum triglyceride levels compared with the consumption of LCT. However, this effect was not seen for the other BMI categories [24]. In our study, participants who were given a combination of butter and MCT oil showed no significant changes in lipoprotein levels. However, the effect of medium-chain fatty acids (MCFAs) on serum lipid levels remains controversial. Recently, Neelakantan et al. reported that coconut oil, a rich source of MCFAs, increases total cholesterol, LDL cholesterol, and HDL cholesterol levels [25]. This may be attributed to the high levels of lauric acid and long-chain SFAs in coconut oil.

Generally, coconut oil does not appear to act differently from other saturated fats on lipoproteins, and a recent systematic review and meta-analysis revealed “no clinically relevant improvement in lipid profile and body composition compared with other oils/fats” [26,27]. McKenzie et al. reported that MCT oil containing 6:0-10:0 MCFAs did not significantly change total cholesterol, LDL-cholesterol, or HDL-cholesterol concentrations, although a small increase in serum triglyceride levels may occur [28]. Thus, the effect of MCT oil on total cholesterol and LDL cholesterol concentrations was dependent on its fatty acid profile. Both the degree of fatty acid saturation and chain lengths of fatty acids determine their effects on lipids. This is supported by previous studies showing that LDL-cholesterol receptor activity and hepatic production rates are independently regulated by saturated and unsaturated fatty acids [29]. Additionally, MCFAs are metabolized through pathways different from those for long-chain SFAs, which may affect cholesterol synthesis [30]. 

While the link between total cholesterol and the risk of coronary artery disease is inconsistent, there is strong evidence linking low-density lipoproteins (especially ApoB) to coronary artery atherosclerosis. The Framingham Offspring Study showed that LDL-p and ApoB outperformed non-HDL in predicting the risk of cardiovascular disease events in metabolic syndrome and diabetes mellitus [31]. Our study showed that for participants with an increased intake of SFAs, no increase in ApoB levels was seen, suggesting that there was no short-term increased risk of coronary atherogenic disease. However, our study was limited by its short duration and the fact that all participants were healthy volunteers. Additionally, we did not study the effect of butter or MCT oil alone on serum lipoproteins because we aimed to investigate the effect of "Bullet coffee" on serum lipids.

Individuals in support of diets high in added SFAs claim that elevated levels of serum cholesterol (due to increased consumption of SFAs) are healthy if HDL cholesterol/HDL-C or cholesterol is high, triglycerides are low, and inflammation and metabolic syndrome are absent [32-34]. A study in 70-year-old individuals did not show an association between total and HDL cholesterol and morbidity and mortality from CHD and all-cause mortality, and these findings support the theory of the beneficial effects of consuming high SFAs [35]. Our study showed that the use of MCT oil and butter did not lower HDL levels, nor did it elevate triglyceride levels. However, a review of recent studies showed that a marked inter-individual variation in LDL responsiveness confounded the relationship between differing sources of SFAs and ASCVD, thus undermining the interpretation of population-based correlations and dietary guidelines [34]. It has also been noted that some individuals have an LDL hyper-response to low carbohydrate “ketogenic” diets often high in SFA [35-37], and these inter-individual responses need to be considered in diet response studies.

There were several limitations in our study. We only enrolled 19 subjects in the control and 22 in the experimental group. Although these were the minimum sample size, a larger number of subjects with age groups ranging from 20 to 50 years would have provided more consistent data. Furthermore, our study subjects mainly comprised Caucasians and African Americans, and including Asian and Hispanic subjects would have yielded additional meaningful data. In the present study, the effects of "Bulletproof coffee" on serum lipids were tested after 12 weeks, although a longer period may have provided the results of the long-term effects of "Bulletproof coffee" on serum lipids. Additional studies involving a control group with coffee and Kerrygold® butter, and another control group with coffee and MCT oil may have yielded interesting results. Studies involving patients with hyperlipidemia may have provided information regarding the possible adverse effects of butter coffee on these groups of patients. However, despite these limitations, our study of “Bulletproof coffee” yielded useful information.

## Conclusions

Our study investigated the effects of “Bulletproof coffee”, a combination of grass-fed butter and MCT oil, on serum lipid levels in healthy adults. The study group consisted of 22 subjects and there were 19 subjects in the control group. Anthropometric variables, detailed serum lipid profile, and thyroid functions were measured at baseline and after 12 weeks. There were no statistically significant differences between the control group and the study group on either occasion. Finally, our study suggests that adding MCT oil and butter to coffee might be safe since no adverse changes in the cholesterol levels were seen after an increase in SFA intake for 12 weeks. However, this study was limited by its relatively short duration and the use of healthy volunteers. A longer-term study with a larger sample size is required to determine the long-term outcomes of the addition of MCT oil and butter to coffee on serum lipids.

## References

[1] Siri-Tarino PW, Sun Q, Hu FB, Krauss RM (2010). Meta-analysis of prospective cohort studies evaluating the association of saturated fat with cardiovascular disease. Am J Clin Nutr.

[2] Astrup A, Magkos F, Bier DM (2020). Saturated fats and health: a reassessment and proposal for food-based recommendations: JACC State-of-the-Art Review. J Am Coll Cardiol.

[3] Zhu Y, Bo Y, Liu Y (2019). Dietary total fat, fatty acids intake, and risk of cardiovascular disease: a dose-response meta-analysis of cohort studies. Lipids Health Dis.

[4] (2024). 2010 Dietary Guidelines, Office of Disease Prevention and Health Promotion. http://www.health.gov/dietaryguidelines/2010.asp.

[5] Lichtenstein AH, Appel LJ, Vadiveloo M (2021). 2021 Dietary Guidance to Improve Cardiovascular Health: a scientific statement from the American Heart Association. Circulation.

[6] Jakobsen MU, O'Reilly EJ, Heitmann BL (2009). Major types of dietary fat and risk of coronary heart disease: a pooled analysis of 11 cohort studies. Am J Clin Nutr.

[7] Hooper L, Martin N, Jimoh OF, Kirk C, Foster E, Abdelhamid AS (2020). Reduction in saturated fat intake for cardiovascular disease. Cochrane Database Syst Rev.

[8] Kris-Etherton PM, Derr J, Mitchell DC (1993). The role of fatty acid saturation on plasma lipids, lipoproteins, and apolipoproteins: I. Effects of whole food diets high in cocoa butter, olive oil, soybean oil, dairy butter, and milk chocolate on the plasma lipids of young men. Metabolism.

[9] Hooper L, Summerbell CD, Thompson R, Sills D, Roberts FG, Moore H, Davey Smith G (2011). Reduced or modified dietary fat for preventing cardiovascular disease. Cochrane Database Syst Rev.

[10] Borén J, Chapman MJ, Krauss RM (2020). Low-density lipoproteins cause atherosclerotic cardiovascular disease: pathophysiological, genetic, and therapeutic insights: a consensus statement from the European Atherosclerosis Society Consensus Panel. Eur Heart J.

[11] Mach F, Baigent C, Catapano AL (2020). 2019 ESC/EAS guidelines for the management of dyslipidaemias: lipid modification to reduce cardiovascular risk. Eur Heart J.

[12] Handelsman Y, Jellinger PS, Guerin CK (2020). Consensus Statement by the American Association of Clinical Endocrinologists and American College of Endocrinology on the Management of Dyslipidemia and Prevention of Cardiovascular Disease Algorithm - 2020 executive summary. Endocr Pract.

[13] St-Onge MP, Jones PJ (2003). Greater rise in fat oxidation with medium-chain triglyceride consumption relative to long-chain triglyceride is associated with lower initial body weight and greater loss of subcutaneous adipose tissue. Int J Obes Relat Metab Disord.

[14] Tsuji H, Kasai M, Takeuchi H, Nakamura M, Okazaki M, Kondo K (2001). Dietary medium-chain triacylglycerols suppress accumulation of body fat in a double-blind, controlled trial in healthy men and women. J Nutr.

[15] St-Onge MP, Jones PJ (2002). Physiological effects of medium-chain triglycerides: potential agents in the prevention of obesity. J Nutr.

[16] Baumeister A, Gardemann J, Fobker M, Spiegler V, Fischer T (2021). Short-term influence of caffeine and medium-chain triglycerides on ketogenesis: a controlled double-blind intervention study. J Nutr Metab.

[17] Elwood PC, Pickering JE, Givens DI, Gallacher JE (2010). The consumption of milk and dairy foods and the incidence of vascular disease and diabetes: an overview of the evidence. Lipids.

[18] Chen M, Sun Q, Giovannucci E, Mozaffarian D, Manson JE, Willett WC, Hu FB (2014). Dairy consumption and risk of type 2 diabetes: 3 cohorts of US adults and an updated meta-analysis. BMC Med.

[19] Zhou D, Yu H, He F (2014). Nut consumption in relation to cardiovascular disease risk and type 2 diabetes: a systematic review and meta-analysis of prospective studies. Am J Clin Nutr.

[20] Iggman D, Risérus U (2011). Role of different dietary saturated fatty acids for cardiometabolic risk. Clin Lipidol.

[21] Toklu B, Milne V, Bella M, Underberg JA (2015). Rise in serum lipids after dietary incorporation of “Bulletproof coffee”. J Clin Lipidol.

[22] Werner LB, Hellgren LI, Raff M, Jensen SK, Petersen RA, Drachmann T, Tholstrup T (2013). Effects of butter from mountain-pasture grazing cows on risk markers of the metabolic syndrome compared with conventional Danish butter: a randomized controlled study. Lipids Health Dis.

[23] Khaw KT, Sharp SJ, Finikarides L, Afzal I, Lentjes M, Luben R, Forouhi NG (2018). Randomised trial of coconut oil, olive oil or butter on blood lipids and other cardiovascular risk factors in healthy men and women. BMJ Open.

[24] Zhang Y, Liu Y, Wang J (2010). Medium- and long-chain triacylglycerols reduce body fat and blood triacylglycerols in hypertriacylglycerolemic, overweight but not obese, Chinese individuals. Lipids.

[25] Neelakantan N, Seah JY, van Dam RM (2020). The effect of coconut oil consumption on cardiovascular risk factors: a systematic review and meta-analysis of clinical trials. Circulation.

[26] Sekhar S, Makaram Ravinarayan S, Kashmer D Yu A (2022). Are we nuts over coconuts? Studying the effects of coconut oil on low-density lipoprotein and cardiovascular diseases: a systematic review. Cureus.

[27] Duarte AC, Spiazzi BF, Zingano CP (2022). The effects of coconut oil on the cardiometabolic profile: a systematic review and meta-analysis of randomized clinical trials. Lipids Health Dis.

[28] McKenzie KM, Lee CM, Mijatovic J, Haghighi MM, Skilton MR (2021). Medium-chain triglyceride oil and blood lipids: a systematic review and meta-analysis of randomized trials. J Nutr.

[29] Mensink RP, Zock PL, Kester AD, Katan MB (2003). Effects of dietary fatty acids and carbohydrates on the ratio of serum total to HDL cholesterol and on serum lipids and apolipoproteins: a meta-analysis of 60 controlled trials. Am J Clin Nutr.

[30] Mensink Mensink, Ronald P. & World Health Organization. (‎2016 (2024). World Health Organization: Effects of saturated fatty acids on serum lipids and lipoproteins: a systematic review and regression analysis. https://iris.who.int/handle/10665/246104.

[31] Cromwell WC, Otvos JD, Keyes MJ (2007). LDL particle number and risk of future cardiovascular disease in the Framingham Offspring Study - implications for LDL management. J Clin Lipidol.

[32] Danesh J, Whincup P, Walker M (2000). Low grade inflammation and coronary heart disease: prospective study and updated meta-analyses. BMJ.

[33] Hansson GK (2005). Inflammation, atherosclerosis, and coronary artery disease. N Engl J Med.

[34] Krumholz HM, Seeman TE, Merrill SS (1994). Lack of association between cholesterol and coronary heart disease mortality and morbidity and all-cause mortality in persons older than 70 years. JAMA.

[35] Griffin BA, Mensink RP, Lovegrove JA (2021). Does variation in serum LDL-cholesterol response to dietary fatty acids help explain the controversy over fat quality and cardiovascular disease risk?. Atherosclerosis.

[36] Goldberg IJ, Ibrahim N, Bredefeld C (2021). Ketogenic diets, not for everyone. J Clin Lipidol.

[37] Norwitz NG, Mindrum MR, Giral P (2022). Elevated LDL-cholesterol levels among lean mass hyper-responders on low-carbohydrate ketogenic diets deserve urgent clinical attention and further research. J Clin Lipidol.

